# Simultaneous Characterization of Metabolic, Cardiac, Vascular and Renal Phenotypes of Lean and Obese SHHF Rats

**DOI:** 10.1371/journal.pone.0096452

**Published:** 2014-05-15

**Authors:** Gina Youcef, Arnaud Olivier, Clément P. J. L'Huillier, Carlos Labat, Renaud Fay, Lina Tabcheh, Simon Toupance, Rosa-Maria Rodriguez-Guéant, Damien Bergerot, Frédéric Jaisser, Patrick Lacolley, Faiez Zannad, Laurent Vallar, Anne Pizard

**Affiliations:** 1 UMRS U1116 Inserm, Vandoeuvre-lès-Nancy, France; 2 Fédération de Recherche 3209, Nancy, France; 3 Université de Lorraine, Nancy, France; 4 Genomics Research Unit, Centre de Recherche Public de la Santé, Strassen, Luxembourg; 5 CHU Nancy, Nancy, France; 6 CIC 1433, Pierre Drouin, Vandoeuvre-lès-Nancy, France; 7 UMR 7365 CNRS, Vandoeuvre-lès-Nancy, France; 8 U954 Inserm, Vandoeuvre-lès-Nancy, France; 9 CIC 9201, PARCC, HEGP, Paris, France; INRA, France

## Abstract

Individuals with metabolic syndrome (MetS) are prone to develop heart failure (HF). However, the deleterious effects of MetS on the continuum of events leading to cardiac remodeling and subsequently to HF are not fully understood. This study characterized simultaneously MetS and cardiac, vascular and renal phenotypes in aging Spontaneously Hypertensive Heart Failure lean (SHHF^+/?^ regrouping ^+/+^ and ^+/cp^ rats) and obese (SHHF^cp/cp^, “cp” defective mutant allele of the leptin receptor gene) rats. We aimed to refine the milestones and their onset during the progression from MetS to HF in this experimental model. We found that SHHF^cp/cp^ but not SHHF^+/?^ rats developed dyslipidemia, as early as 1.5 months of age. This early alteration in the lipidic profile was detectable concomitantly to impaired renal function (polyuria, proteinuria but no glycosuria) and reduced carotid distensibility as compared to SHHF^+/?^ rats. By 3 months of age SHHF^cp/cp^ animals developed severe obesity associated with dislipidemia and hypertension defining the onset of MetS. From 6 months of age, SHHF^+/?^ rats developed concentric left ventricular hypertrophy (LVH) while SHHF^cp/cp^ rats developed eccentric LVH apparent from progressive dilation of the LV dimensions. By 14 months of age only SHHF^cp/cp^ rats showed significantly higher central systolic blood pressure and a reduced ejection fraction resulting in systolic dysfunction as compared to SHHF^+/?^. In summary, the metabolic and hemodynamic mechanisms participating in the faster decline of cardiac functions in SHHF^cp/cp^ rats are established long before their physiological consequences are detectable. Our results suggest that the molecular mechanisms triggered within the first three months after birth of SHHF^cp/cp^ rats should be targeted preferentially by therapeutic interventions in order to mitigate the later HF development.

## Introduction

Chronic heart failure (HF) is of heterogeneous etiology but it usually occurs in the elderly [1] and unlike other cardiovascular problems its prevalence is increasing. Several studies demonstrated that patients affected by metabolic syndrome (MetS) -defined as the simultaneous occurrence of at least three of the five following risk factors: obesity, hypertension, dyslipidemia, type 2 diabetes and insulin resistance- have a higher risk of developing HF [2], [3], [4], [5]. Indeed, it has been described that long-lasting hypertension induces left ventricular hypertrophy (LVH) and later dilatation of the LV internal cavity [6]. Diabetes as well as insulin resistance have been also associated with ventricular dysfunction and increased heart mass [7]. Furthermore, the risk of developing chronic HF has been demonstrated to be higher in obese patients and more specifically so, in those presenting abdominal obesity, a fundamental feature of MetS [8], [9], [10]. Thus, metabolic risk appears to be critical for the development of HF suggesting that the rising incidence of HF over the last decade probably mirrors the concurrent epidemics of obesity. Identifying and managing the patients at risk of HF prior to the onset of symptoms may be an effective approach to prolong active life by delaying or preventing the onset of HF in those patients [11]. For this attractive preventive strategy to be effective, a deep understanding of the continuum of events underpinning the transition from obesity/MetS to HF is required. Furthermore a better characterization of risk factors for the transition to HF is likely to provide new preventive therapeutic opportunities.

To study the influence of MetS on the cardiac, renal and vascular remodeling leading to the development of HF, an experimental animal model such as the Spontaneously Hypertensive rats prone to HF (SHHF/MccGmiCrl- Lepr^cp^, SHHF) might be particularly relevant [12], [13].

Obtained from the seventh backcross between SHR/N-cp obese and SHR/N rats (Spontaneously hypertensive) [14], the SHHF rat model is of great interest in our study since it mimics the same pathophysiology of human MetS and HF [12], [13], [15], [16], [17], [18].

To our knowledge, it represents the only available model that could progressively and spontaneously develop HF with earlier onset when rats are homozygous for the mutant allele of the leptin receptor gene Lepr^cp^, a genotype that renders them hyperphagic and obese [19]. Interestingly, the onset of HF varies accordingly to the Lepr^cp^ allele dosage and consequently obese homozygous mutant animals SHHF^cp/cp^ die before the lean littermates (heterozygous, SHHF^+/cp^ and wild type, SHHF^+/+^ later referred to as SHHF^+/?^) [12].

Although some studies have been published using the SHHF rat model, the data are too scattered to draw clear-cut conclusions on the continuum of events leading to HF. Indeed, if reports have focused either on the SHHF cardiac [15], [20], [21] or renal features [22] and the combination of both is rarely addressed. As they are not based on the concurrent observation of obese versus lean animals in similar conditions (sex, diet, analyzed parameters), conclusions from these studies are difficult to combine in order to get a unified model. Furthermore, the vascular phenotypes of the obese and their control lean SHHF rats have not yet been evaluated.

The present study has been therefore designed to provide an integrated status of the metabolic, cardiac and vascular phenotypes of the SHHF obese as compared to lean rats in order to fully appreciate the impact of MetS on the progression towards HF.

## Materials and Methods

### Animal model

Male 1 month-old lean (homozygous wild type SHHF^+/+^ and heterozygous SHHF^+/cp^, hereafter referred to as SHHF^+/?^, n = 23) and obese (SHHF^cp/cp^, n = 21) Spontaneously Hypertensive Heart Failure rats (SHHF/MccGmiCrl-Lepr^cp^) were purchased from Charles River Laboratories (L'Arbresle, France). Animals were maintained on a 12:12-h light-dark cycle with *ad libitum* access to tap water and modestly enriched fatty diet (Purina 5008, Charles River, France) as recommended by the supplier. The composition of the diet by weight was 23% protein, 58.5% carbohydrate, 6.5% fat (as compared to 3.15% in standard diet; M20 SDS)), 4.0% fiber and 8.0% ash. Delivery of calories from each component was: approximately 16.7% from fat, 26.8% from proteins and 56.4% from carbohydrates. After a 2-week-period of acclimation, fourteen 1.5 month-old SHHF^+/?^ and twelve SHHF^cp/cp^ underwent detailed metabolic, cardiac, renal and vascular phenotyping before being euthanized by exsanguination leading to cardiac arrest. The remaining animals were monitored regularly over a period of 12.5 months in order to evaluate their metabolic, cardiac, renal and vascular status over time. Experimental protocols were carried out according to the institutional animal care and use committee of Inserm in order to minimize animal suffering. This protocol was reviewed and the experiments were monitored by the staff of the animal facility, which was authorized (agreement # B54-547-17) by the Ministry and local authorities.

Three SHHF^cp/cp^ rats died while none of the SHHF^+/?^ did during the confinement at 37°C required for the phethymography blood pressure measuring experiment. One SHHF^cp/cp^ rat was found dead in his cage while no confounding behaviour warned us of such a potential event. Although those sudden deaths suggest a fragility of the SHHF^cp/cp^ rats as compared to their lean counterparts it seemed inappropriate to draw a Kaplan Meier curve while most of the deaths occurring within the group of obese rats intervened during their phenotyping. Thus, the end point of our study (month 14) was determined by the number of SHHF^cp/cp^ rats still alive that had to be sufficient to allow statistical comparison with the SHHF^+/?^ group of rats.

### Metabolic cages

All animals were individually transferred into siliconed metabolic cages (Silicone Solution in Isopropanol, SERVA; Tecniplast, France) where they were given ad libitum access to water and powdered food (Purina 5008, Charles River). Animals were followed during three consecutive days with daily monitoring of their water and food consumption as well as collection of fecal and urinary excretion. The intakes and excretions were estimated during the 3-day follow-up by averaging daily measurements to mitigate the possible changes linked to rat acclimation to the metabolic cages. Urine samples were collected on a daily basis and centrifuged at 4,500 rpm for 10 min at RT in order to discard cell debris and other solid materials before being stored at −20°C for further biochemical analysis. Urinary osmolality, ionogram, enzymatic creatinine and aldosterone levels were determined in urine samples collected on the third day since it corresponds to a physiological steady state of the rats. Osmolality was assayed using a freezing point osmometer (Roebling, Germany) whereas creatinine and aldosterone were measured with commercial kits according to the manufacturer's recommendations (OSR61204, Beckman Coulter and Siemens 06615154 Coat-A-Count RIA Aldosterone Kit, respectively).

To evaluate renal performance and activity, glomerular filtration rate (eGFR) was estimated in ml/24h and calculated as follows:

**eGFR** (ml/24 h)  =  [(Urinary creatinine concentration (mmol/L) × Urinary daily volume (mL/24 h))/serum creatinine concentration (mmol/L)].

### Echocardiography

Transthoracic echocardiography was performed on anesthetized rats (Isoflurane 5% initial, 3% maintenance, in 1.5 L/min dioxygen) at different time points: 1.5, 3, 6, 9, 12 and 14 months in the left decubitus position using a 12 MHz pediatric transducer connected to a Sonos 5500 Ultrasound System (Philips, France). Short axis M-mode echocardiograms were obtained for measurement of Left Ventricle (LV) Internal Diameters at end diastole (LVIDd) and end systole (LVIDs), LV Fractional Shortening (FS), Ejection Fraction (EF), Septal (Septum) and Posterior Wall thickness (PWT). Doppler flow velocities were taken at the level of the mitral valve in the apical four-chamber view with the Doppler probe placed at the edge of the mitral leaflets where the peak of early (E) and late filling waves (A) as well as E wave deceleration time (EDT) were measured. Measurements and calculations used were as follows:

- **FS** (%)  =  [(LVIDd – LVIDs)/LVIDd] ×100.
- **EF** (%)  =  [(EDV- ESV)/EDV] ×100 where EDV and ESV referred to End-diastolic and End-systolic volumes respectively.
- **LV mass** (mg)  = 1.04× [(PWT+Septum+LVIDd)^3^-(LVIDd)^3^] where 1.04 is the specific gravity of the myocardium.

All echocardiographic parameters were calculated by averaging results from three to four consecutive cardiac cycles for each rat at each time point.

### Blood Pressure measurements

Peripheric Systolic blood pressure (SBP) as well as HR were monitored at different time points in conscious animals using the tail-cuff method (Visitech BP-2000 Systems, France). After the placement of the rats in a warmed chamber to allow caudal vein dilatation, the tail-cuff computerized non-invasive method was set to measure 10 preliminary cycles followed by 10 effective measurements. To prevent bias in data due to animal anxiety, all rats were familiarized with the procedure for at least one week before measurements.

### Invasive Blood pressure and Arterial stiffness measurements

Arterial diameter (right carotid artery) and central blood pressure (diastolic, systolic and pressure pulse (PP) on left carotid artery) were simultaneously recorded in isoflurane-anesthetized rats (3% of isoflurane in 1.5 L/min dioxygen). Internal arterial diameter was measured using a 17 MHz ultrasonic echo-tracking device (NIUS-01; Asulab SA, Marin, Switzerland). 1.5- and 14-month-old rats were characterized for their carotid distensibility and compliance as well as the incremental elastic modulus (Einc) and circumferential wall stress (σ) as described previously [23].

### Histology

After arterial hemodynamic property evaluation, anesthetized animals were euthanized by exsanguination leading to cardiac arrest and organs were rapidly dissected and weighted. Organs and tissue specimens were washed in physiological saline, fixed in formalin for 24 h, and then preserved in 70% ethanol before being paraffin-embedded. Histological slides for heart, kidney, liver and peri-renal visceral adipose tissue were prepared from 5 µm thick sections of the paraffin blocks and stained using Sirius Red or hematoxylin-eosin. Myocardial fibrosis was determined on Sirius red stained heart sections by measuring the percentage of fibrotic area to whole heart section area using Image J software. Paraffin-embedded carotid sections underwent Weigert's staining in order to measure the carotid mean cross sectional area (MCSA) as previously described [23]. Glomerular surface area was traced manually on different places on the kidney section and measured by using NIS-element software (Nikon). Results were the average of up to 50 glomeruli per animal of each group.

### Blood collection and Biochemical assays

Arterial blood samples were collected at 1.5 months and 14 months of age through a catheter implanted in the carotid of the anesthetized rats. Venous blood samples were collected from the jugular vein puncture at several time points taking advantage of the anaesthesia of the rats that underwent echocardiographic examination. Plasma samples were collected in presence of sodium citrate (1∶10 v:v of blood) to separate plasma after immediate centrifugation at 3,000 rpm for 10 min at room temperature (RT).

Plasma samples from 1.5 and 14 months old rats were used to evaluate Brain Natriuretic Peptide 45 (BNP 45) using a BNP 45 Rat ELISA Kit (Abcam, ab108816) according to manufacturer's instructions.

Serum samples were collected after 20 min sedimentation at RT and subsequent 2,300-rpm centrifugation for 15 min. Serum samples from 16 h-fasted rats at 1.5 and 14 months of age were used to evaluate total cholesterol, triglycerides, free fatty acids, creatinine and glucose concentrations by routine enzymatic methods on an automatic biochemical analyzer, sodium and potassium (Na^+^, K^+^) by a standardized indirect potentiometry technique. Adiponectin and Insulin levels were measured using commercially available kits according to manufacturer's instructions of the Adiponectin Rat ELISA Kit (Abcam, #ab108784) and Insulin Human ELISA kit (Abcam, #ab100578) respectively.

Insulin resistance (IR) was evaluated in rats using the homeostasis model assessment (HOMA) by calculating the HOMA-IR index as follows:

HOMA-IR  =  [fasting glucose concentration (mmol/L) × fasting insulin concentration (µIU/mL)]/22.5.

### Statistics

All results are presented as mean ± sem. Statistical analysis of data was performed using unpaired Student's t test to compare the genotypes and ages respectively with *p<0.05, **p<0.01, ***p<0.001 and ^§^p<0.05, ^§§^p<0.01, ^§§§^p<0.001 to be considered statistically significant. Non-parametric ANOVA analysis with two factors allowed the evaluation of interaction between aging and genotypes.

## Results

### Early Metabolic disorders in SHHF^cp/cp^ rats

**Figure 1:**
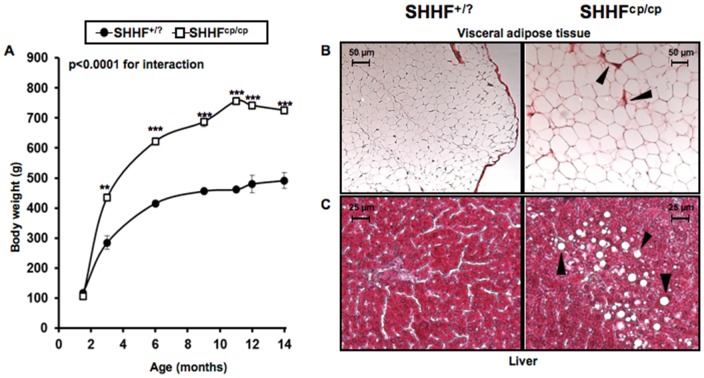
Metabolic follow-up. **A**- The monitoring of body weight shows that the onset of obesity occurs during the first three months after birth of SHHF^cp/cp^ rats. Progressively, the SHHF^cp/cp^ rats continue to gain weight accentuating their differences with the SHHF^+/?^ (n = 5 to 14 rats per genotype). (**B–C**) Paraffin embedded tissues dissected from SHHF^cp/cp^ and SHHF^+/?^ rats at 14 months of age showing metabolic disorder related-tissue alterations **B**- Peri-renal visceral fat of SHHF^cp/cp^ rats stained with Sirius red exhibited marked fibrosis (arrows) and hypertrophic adipocytes. **C**- Hemaetoxylin & Eosin staining shows the deposition of lipid droplets (arrows) in the liver dissected from SHHF^cp/cp^ rats suggesting the development of non-alcoholic hepatic steatosis. Pictures are representative of each analyzed group (n = 5 to 7 rats per genotype). Values are mean ± sem. Non-parametric ANOVA analysis with two factors allowed the evaluation of an interaction between aging and genotype. * p<0.05, ** p<0.01, *** p<0.001 for comparing SHHF^cp/cp^ vs. SHHF^+/?^ at the same time point.

While no differences were observed at 1.5 months of age, SHHF^cp/cp^ gained significantly more weight during the following 3 months than their SHHF^+/?^ littermates as animals underwent a rapid growth phase (Figure 1A). Then, both groups of rats showed a slower and almost parallel growth phase. At 14 months of age, SHHF^cp/cp^ and SHHF^+/?^ rat weight increased by about 6- and 4- fold, respectively (Figure 1A). Histological analysis of peri-renal visceral fat revealed the presence of very large adipocytes and evidence of fibrosis in SHHF^cp/cp^ rats as compared to that of SHHF^+/?^ rats (Figure 1B).

Altered plasma metabolic profiles were detectable in SHHF^cp/cp^ rats as early as 1.5 months of age. These included higher levels of total cholesterol, HDL cholesterol, free fatty acids (FFA) and triglycerides (TG) (Table 1). The overall increase in blood lipid concentration in the SHHF^cp/cp^ rats was significantly maintained over the time leading to major dyslipidemia at 14 months of age (Table 1). While the fasting glycemia levels were not modified either over time or between genotypes, fasting insulin levels increased significantly in animal of both genotypes but more dramatically in the SHHF^cp/cp^ group indicating the development of an insulin resistance (IR) (Table 1). The IR development was confirmed by the HOMA-IR index, that discriminated the SHHF^cp/cp^ rats from the SHHF^+/?^ as early as 1.5 months of age. Adiponectin levels were higher as early as 1.5 months of age in the homozygous mutant group. At both ages, plasma BNP concentrations were not different between genotypes but significantly increased over time in SHHF^cp/cp^ and SHHF^+/?^ rats. No differences were recorded in serum sodium and potassium levels between SHHF^cp/cp^ and SHHF^+/?^ rats throughout the follow-up (Table 1). Hepatic steatosis was detected only at 14 months of age in livers dissected from SHHF^cp/cp^ rats (Figure 1C).

**Table 1:** Blood parameters.

Genotype	SHHF^+/?^	SHHF^cp/cp^	SHHF^+/?^	SHHF^cp/cp^		ANOVA		
Parameters								
Age, month	1.5	1.5	14	14	Genotype	Age	Interaction	
**N**	7–12	5–10	7	5				
**Ionogram**, mmol/l								
Na^+^	140.8±0.5	141.4±0.6	143.7±0.7**^§§§^**	143.0±0.8	ns	0.0003	ns	
K^+^	4.8±0.2	4.9±0.2	5.3±0.2	4.7±0.2	ns	ns	ns	
Na^+^/K^+^ ratio	29.8±0.9	28.8±1.0	28.1±1.2	30.3±1.4	ns	ns	ns	
**Lipidic profil**, g/l								
Total Cholesterol	0.7±0.2	0.9±0.3^***^	0.8±0.3	4.6±0.4^***§§§^	<0.0001	<0.0001	<0.0001	
HDL	0.2±0.0	0.3±0.0^**^	0.2±0.0	0.4±0.0^***§§§^	<0.0001	<0.0001	<0.0001	
LDL	0.4±0.1	0.5±0.1	0.4±0.1	1.3±0.2**^§^**	0.0026	0.0045	0.0141	
FFA	1.1±0.4	1.7±0.5^*^	1.1±0.5	5.2±0.6^*§^	<0.0001	0.0045	0.0023	
TG	0.3±0.7	0.8±0.0^*^	0.5±0.1	14.6±1.1^***§§§^	<0.0001	<0.0001	<0.0001	
**Fasting glycemia**, mg/dl	83±5	100±12	107±5	92±9	ns	ns	ns	
**Fasting insulin**, µIU/ml	12±2	25±4	16±4**^§§^**	226±92^***§§§^	0.007	0.004	0.009	
**HOMA-IR index**	0.9±0.3	2.6±0.8^***^	2.0±0.3**^§§§^**	14.6±5.9^***§§^	0.004	0.007	0.022	
**Creatinine**, mmol/l	2.13±0.60	1.88±0.69^**^	4.60±0.79**^§§§^**	8.00±0.93^***§§§^	0.0489	<0.0001	0.0244	
**BNP**, pg/ml	117±18	116±24	212±29**^§§^**	207±29**^§§^**	ns	0.0013	ns	
**Adiponectin**, µg/ml	6.4±0.5	19.7±0.8^***^	5.3±0.7**^§^**	12.8±0.8^***§§§^	<0.0001	<0.0001	0.0007	

**Na^+^**, sodium, **K^+^**, potassium; **HDL**, High Density Lipoprotein; **LDL**, Low density Lipoprotein; **FFA**, Free Fatty Acids; **TG**, triglyceride; **BNP**, Brain Natriuretic Peptide. Values are the mean±sem. Non-parametric ANOVAs analysis with two factors allowed the evaluation of interaction between aging and genotype. Student's T test * p<0.05; ** p<0.01, *** p<0.001 to compare SHHF ^cp/cp^ *vs.* SHHF^+/?^ at same time point; § p<0.05; §§p<0.01, §§§ p<0.001 to compare T14-mo *vs* T1.5-mo for a same genotype; **N** stands for the number of samples; **ns** stands for not significant.

### Worsening of the renal function associated with ^cp/cp^ genotype

**Figure 2:**
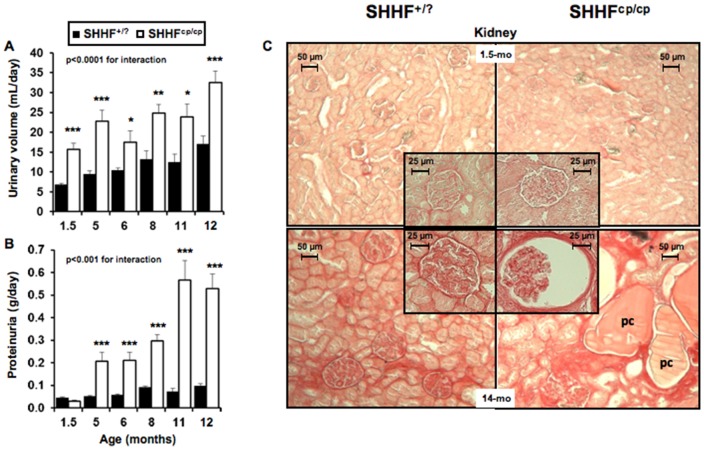
Renal function follow-up. The worsening of renal function associated with the ^cp/cp^ genotype was evaluated while rats were placed individually in metabolic cages for 3 consecutive days (n = 5 to 10 rats per genotype). The alteration of renal function observed in SHHF^cp/cp^ rats is shown by **A**- increased urine excretion as early as 1.5 months of age, **B**- increased proteinuria and **C**- by major deteriorations of renal histological ultrastructure at 14 months for SHHF^cp/cp^ rats ie. massive protein casts (pc), fibrosis, tubular atrophy and enlarged glomerular surfaces (insert in the bottom right panel). Pictures are representative of each analyzed group (n = 5 to 14 rats per genotype); 5-fold magnification for the global kidney picture and 20-fold for the glomeruli (inserts). Values are mean ± sem. Non-parametric ANOVA analysis with two factors allowed the evaluation of an interaction between aging and genotype. * p<0.05, ** p<0.01, *** p<0.001 for comparing SHHF^cp/cp^ vs. SHHF^+/?^ at the same time point.

Average water intake (Table 2) and urine excretion (Figure 2A) estimated over three consecutive days indicated that SHHF^cp/cp^ rats developed polyuria as early as 1.5 months of age before showing evidence of polydipsia. Up to 12 months of age volumes of water consumed and urinary excretion were greater in SHHF^cp/cp^ rats as compared to SHHF^+/?^ animals (Table 2 and Figure 2A, respectively). This occurred concomitantly with a progressive decrease in urine osmolality and creatinine (Table 2), and in parallel with a progressive increase in proteinuria over the animals lifetime measured in the urine obtained on the third day in the metabolic cages (Figure 2B). Interestingly, while showing higher daily sodium and potassium excretions at 1.5 months of age, SHHF ^cp/cp^ rats reduced their excretions over time to concentrations similar to those of the SHHF^+/?^ rats (Table 2). It is noteworthy that the significantly smaller sodium to potassium ratio in SHHF^cp/cp^ at 1.5 months of age was observed while a higher urinary excretion of aldosterone was detected in those rats as compared to the SHHF^+/?^ animals (Table 2).

**Table 2:** Renal parameters.

Genotype	SHHF^+/?^	SHHF^cp/cp^	SHHF^+/?^		SHHF^cp/cp^			ANOVA	
Parameters									
Age, month	1.5	1.5		12	12	Genotype	Age		Interaction
**N**	5–10	6–10		7	5				
**KW**, g	0.64±0.03	0.69±0.01^*^		1.69±0.04^§§§^	1.97±0.05^*^ ^§§§^	0.0007	<0.0001		0.0114
**KW/Tibia length**, g/cm	0.27±0.01	0.32±0.01^**^		0.39±0.02^§§§^	0.49±0.02^**^ ^§§§^	<0.0001	<0.0001		0.0459
**Glomerular surface area**, µm^2^	6179±777	7527±905		14969±1095^§§^	18864±1347^*^ ^§§§^	ns	<0.0001		ns
**Water intake**, ml/24h	21.5±1.8	22.5±1.8		30.7±3.2^§^	41.5±2.9^**§§§^	ns	<0.0001		0.019
**Osmolality**, mOsmol/kg H_2_O	2583±103	1630±120		2268±103^§§§^	986±126^**§§§^	0.002	<0.0001		ns
**Ionogram**, mmol/24h									
Na^+^	1668±191	2639±177^***^		1065±81^§§^	1136±93^§§§^	0.001	<0.0001		0.014
K^+^	2965±321	5685±298^***^		2907±267	2886±289^§§§^	0.0001	0.0009		0.001
Na^+^/K^+^ ratio	0.57±0.02	0.46±0.01^**^		0.38±0.03^§§^	0.38±0.03^§^	0.046	<0.0001		0.012
**Urine Creatinine**, mmol//24h	72.8±5.5	81.0±5.2^***^		115.2±4.7^§§^	66.4±5.1**^§^**	0.0001	0.0434		0.0005
**Aldosterone**, ng/24h	3.66±1.51	14.02±1.78^*^		9.40±0.88^§§§^	7.18±1.01^§§§^	0.0195	ns		0.0002
**eGFR**, mL/24h	35±4	25±2		42±2**^§^**	11±2^***§§§^	ns	<0.0001		0.0004

**KW**, Kidney Weight; **Na^+^**, sodium, **K^+^**, potassium, **eGFR**, estimated Glomerular filtration rate; Values are the mean±sem. Non-parametric ANOVAs analysis with two factors allowed the evaluation of interaction between aging and genotype. Student's T test *, ** and *** p<0.01, p<0.001 and p<0.0001 respectively when comparing SHHF ^+/?^ *vs.* SHHF^cp/cp^ at same time point; § p<0.05, §§ p<0.01 and §§§ p<0.001 to compare T14-mo *vs* T1.5-mo for a same genotype; **N** stands for the number of samples; **ns** stands for not significant.

The kidney weight-to-tibia length ratio was greater in the SHHF^cp/cp^ rats than in the SHHF^+/?^ rats (Table 2). However, histology and tissue architecture of the kidney of the obese rats did not appear altered at 1.5 months of age (Figure 2C). In contrast, abnormal architecture of the kidney was observed in 14 month-old in SHHF^cp/cp^ rats compared to SHHF^+/?^. Renal lesions in obese rats included increases in glomerular surface area (Figure 2C and Table 2, respectively) associated with massive protein casts in the Bowman's space and tubular lumens in both kidney cortex and medulla. In SHHF^+/?^ rats, tissue alterations were restricted mostly to the cortex. At 14 months of age, the abnormal histological modifications were observed simultaneously with a significant reduction of the estimated glomerular filtration rate (eGFR) in the SHHF^cp/cp^ group thus reflecting significantly reduced kidney function (Table 2).

### Exacerbated cardiac remodeling in SHHF^cp/cp^ rats

**Figure 3:**
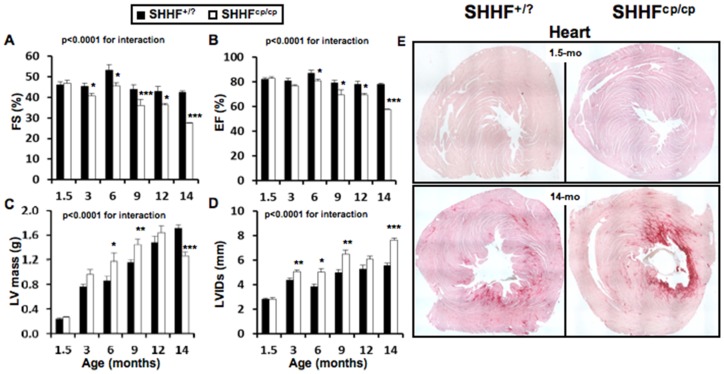
Cardiac follow-up. Transthoracic echocardiograms were performed on isoflurane-anesthetized SHHF at different time points throughout the protocol (1.5; 6; 9 and 14 months of age, n = 5 to 10 rats per genotype). **A**- Fractional Shortening (FS) and **B**- Ejection Fraction (EF) showed the progressive but faster decline of heart systolic function in SHHF^cp/cp^ rats compared to SHHF^+/?^ controls. **C**- LV mass as well as **D**- Left Ventricular (LV) Internal Diameters at end systole (LVIDs) were significantly higher in the SHHF^cp/cp^ group from 6 months and continued to rise till 12 and 14 months of age respectively demonstrating LV hypertrophy and dilation **E**- Red Sirius staining performed on heart sections obtained from SHHF^+/?^ and SHHF^cp/cp^ rats at 1.5 and 14 months of age showed greater myocardial fibrosis in 14-month-old SHHF^cp/cp^ rats compared to SHHF^+/?^ from the same age (n = 5 to 7 rats per genotype). Mean ± sem. Non-parametric ANOVAs analysis with two factors allowed the evaluation of an interaction between aging and genotype. * p<0.05, ** p<0.01, *** p<0.001 for comparing SHHF^cp/cp^ *vs.* SHHF^+/?^ at the same age.

At 1.5 months of age, heart weight-to-tibia length ratios were significantly increased in the SHHF^cp/cp^ group as compared to the SHHF^+/?^ group (Table 3). Cardiac hypertrophy was not associated with gross histological modifications of the heart structure or with functional impairment at that age (Figures 3A–E at 1.5 months of age). By contrast, at 14 months of age, Sirius red staining revealed massive cardiac fibrosis in the SHHF^cp/cp^ rats (Figure 3E; 14 months and table 3; % fibrotic area) which was less apparent in SHHF^+/?^ animals. In the former group of rats, this was observed concomitantly with dramatically altered cardiac function parameters together with elevated HW/tibia length (Table 3).

**Table 3:** Cardiac parameters.

Genotype	SHHF^+/?^	SHHF^cp/cp^	SHHF^+/?^	SHHF^cp/cp^			ANOVA		
Parameters									
Age, month	1.5	1.5	14	14	Genotype	Age		Interaction	
**N**	10	10	7	5					
**Cardiac Morphometry**									
HW, g	0.75±0.03	0.77±0.04	1.95±0.04^§§§^	2.08±0.05^***^ ^§§§^	ns	<0.0001		ns	
Tibia length, cm	2.30±0.03	2.13±0.03^*^	4.31±0.04^§§^	3.98±0.05^***^ ^§§^	<0.0001	<0.0001		0.0538	
HW/Tibia, g/cm	0.32±0.01	0.36±0.01^**^	0.45±0.01§	0.52±0.01^*^§	0.0004	<0.0001		ns	
**Cardiac Remodeling, mm**									
LVIDd	5.6±0.2	5.4±0.1	9.6±.01^§§^	10.7±0.2^***^ ^§§§^	0.013	<0.0001		0.002	
Septum Thickness	1.2±0.0	1.1±0.0^*^	2.4±0.0^§§§^	1.6±0.1^***^ ^§§§^	<0.0001	<0.0001		<0.0001	
Posterior Wall Thickness	0.9±0.0	1.0±0.0	1.6±0.0^§§§^	1.1±0.0^***^ ^§§§^	<0.0001	<0.0001		<0.0001	
% myocardial fibrosis	0.10±0.01	0.12±0.02	1.14±0.56	4.75±1.02^*§^	0.0005	0.0013		0.0372	
**LV Diastolic function**									
A, mm/s	31±4	24±2	41±7	92±25	ns	ns		ns	
E, mm/s	96±5	108±6	101±2	124±7	ns	ns		ns	
E/A	3.2±0.4	4.0±0.9	2.9±0.4	1.5±0.4	ns	ns		ns	
EDT, ms	14±1	14±1	32±1^§§^	20±5^***^ ^§^	<0.0001	<0.0001		<0.0001	

**HW**, Heart Weight; **LV**, Left Ventricle; **LVIDd**, Left Ventricle Internal Diameters at diastole; **E** and **A**, early and late filling waves; **EDT**, E-vel Deceleration Time. These echocardiographic parameters are the mean ± sem of the average of three to four consecutive cardiac cycles for each rat. % of Myocardial fibrosis was determined on Sirius red stained heart sections by measuring the percentage of fibrotic area to whole heart section area using Image J software. Non-parametric ANOVAs analysis with two factors allowed the evaluation of interaction between aging and genotype. Student's T test * p<0.05; ** p<0.01, *** p<0.001 to compare SHHF^+/?^ *vs.* SHHF^cp/cp^ at same time point; ; § p<0.05, §§ p<0.01 and §§§ p<0.001 to compare T14-mo *vs* T1.5-mo for a same genotype; **N** stands for the number of rats; **ns** stands for not significant.

Echocardiographic monitoring from 1.5 to 14 months of age demonstrated the development of worsened and faster cardiac remodeling in the SHHF^cp/cp^ group (Table 3 and Figure 3) as compared to the SHHF^+/?^ rats. Indeed, while showing indistinguishable echocardiographic parameters at 1.5 months of age, the two groups of rats developed progressively distinctive features from 6 months of age. The measured cardiac remodeling was indicative of alterations of the cardiac systolic function in the SHHF^cp/cp^ group only (Table 3 and Figure 3A–B). Diastolic function was not different between lean and obese rats at 14 months of age (Table 3; LV Diastolic function).

Interestingly, a significantly heavier mass of the left ventricle (LV) of the SHHF^cp/cp^ was observed as early as 6 months of age, a difference that was sustained through to 9 months of age (Figure 3C). A thinning of the LV wall (Table 3; septum and posterior wall thickness) was present in the SHHF^cp/cp^ that led to dilation as early as 3 months of age (Figure 3D) and the decline by 14 months of age of the left ventricular systolic function (Figure 3A–B). Over the whole period of monitoring, the observed hypertrophic remodeling was of the eccentric type in the SHHF^cp/cp^ animals since their LV dilated and their LV walls had firstly thickened before becoming thinner at 14 months of age (Figure 3C). Meanwhile the SHHF^+/?^ rats developed concentric hypertrophic remodeling as their cardiac LV wall continued to thicken (Table 3; septum and posterior wall thickness and Figure 3C).

### Higher Systolic and pulse pressures in SHHF^cp/cp^ rats

**Figure 4:**
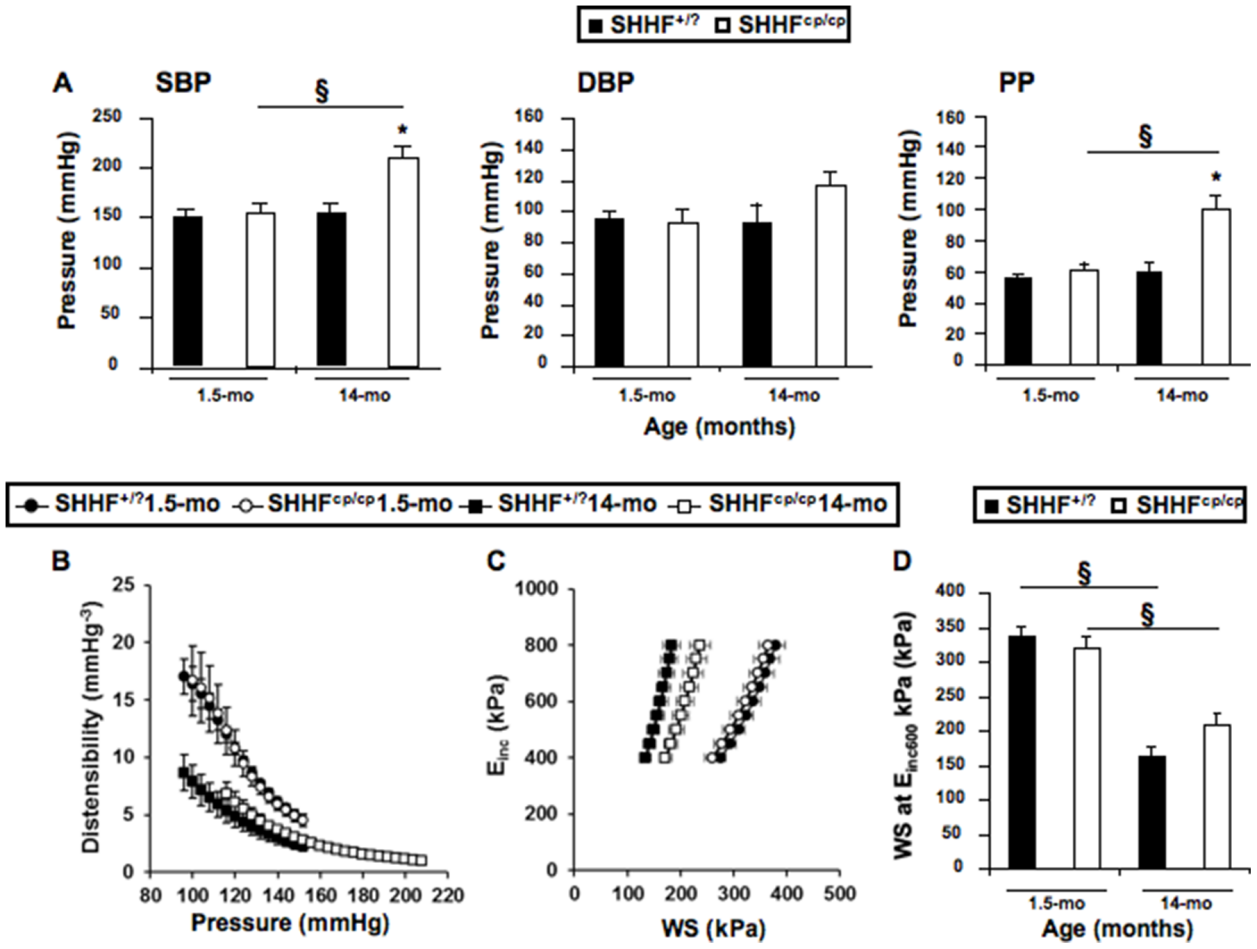
Hemodynamic phenotyping. Invasive blood pressure measurements were obtained on anesthetized rats during vascular phenotyping of the animals. **A**- Systolic (SBP), Diastolic (DBP) Blood Pressures and Pulse Pressure (PP) were measured at both the earliest and latest ages. Results showed that over time only SHHF^cp/cp^ rats increased their SBP and PP becoming significantly higher at 14 months of age compared to SHHF^+/?^ animals. **B**- Distensibility, **C**- Incremental Elastic modulus (E_inc_) to Wall Stress (WS) curves and **D**- WS at Einc 600 kPa were recorded. Values are mean ± sem of 5 to 14 measurements depending on the genotype and age. Fisher's LSD Multiple-Comparison Test * p<0.05 for comparing of SHHF^cp/cp^ *vs.* SHHF^+/?^ rats at the same age; § p<0.05 for comparison of 14 month-old *vs.* 1.5-month-old rats of the same genotype.

Blood pressure measurement was performed either invasively in anesthetized animals at the earliest and latest ages (Table 4) or in conscious rats by plethysmography at different time points (Table 5). We observed that rats of both genotypes presented comparable values of systolic, diastolic and pulse blood pressure (SBP, DBP and PP respectively) at 1.5 months of age, indicating a similar pre-hypertensive state (Figure 4A). The progression towards severe hypertension was monitored in both SHHF^+/?^ and SHHF^cp/cp^ groups of vigil rats by repeated measures of blood pressure using noninvasive phlethysmography (Table 5). Further hemodynamic evaluation using invasive technique on anesthetized rats showed that the central SBP and PP became significantly higher at 14 months of age only for the SHHF^cp/cp^ rats as compared to their SHHF^+/?^ counterparts (Figure 4A).

**Table 4:** Mechanical properties of the carotid artery.

Genotype	SHHF^+/?^	SHHF^cp/cp^	SHHF^+/?^	SHHF^cp/cp^		ANOVA	
Parameters							
Age, month	1.5	1.5	14	14	Genotype	Age	Interaction
**N**	14	12	7	5			
**Diameter**, mm							
Diastolic	0.78±0.02	0.80±0.05	083±0.05	0.96±0.04^§^	ns	0.03	ns
Systolic	1.01±0.02	0.99±0.05	1.01±0.03	1.13±0.05	ns	ns	ns
Mean Blood Pressure, mmHg	113±5	113±8	114±13	150±9	0.046	0.045	0.047
**Values at the MBP**							
Diameter, mm	0.88±0.03	0.88±0.05	0.92±0.04	1.05±0.04^§^	ns	0.046	ns
Compliance, mm^2^/mmHg^−1^.10^3^	5.88±0.29	4.15±0.26^*^	4.45±0.53**^§^**	2.79±0.40^*§^	0.0002	0.002	ns
Distensibility, mm.Hg^−3^	9.98±0.71	7.33±0.73^*^	7.25±1.26**^§^**	2.95±0.38^*§^	0.002	0.002	ns
Einc, kPa	388±58	577±110	421±125	1004±133^*^	0.002	0.049	0.08
WS, kPa	234±28	245±40	142±26	242±35	ns	ns	ns
MCSA, mm^2^	0.084±0.013	0.087±0.019	0.149±0.014^§^	0.132±0.060^§^	ns	<0.00001	ns

**MBP**, Mean Blood Pressure; **Einc**, Incremental Elastic-modulus; **WS**, Wall Stress; **MCSA**, Mean Cross Sectional Area. Values are mean ± sem. Non-parametric ANOVAs analysis with two factors allowed the evaluation of interaction between aging and genotype. Fisher's LSD Multiple-Comparison Test * p<0.05 to compare SHHF^cp/cp^ *vs.* SHHF^+/?^ at the same age; § p<0.05 to compare of 14 month-old vs. 1.5-month-old rats of the same genotype; **N** stands for the number of rats; **ns** stands for not significant.

**Table 5:** Blood pressure follow-up in conscious SHHF rats.

Genotype	SHHF^+/?^				SHHF^cp/cp^				ANOVA			
Age, month	2.5	5	13	14	2.5	5	13	14	Genotype	Age	Interaction	
**N**	8	8	7	7	9	9	6	5				
**SBP**, mmHg	176±4	182±12	187±6	195±8	150±8	163±14	182±7	207±9	ns	<0.0001	ns	
**HR**, bpm	397±5	383±12	466±13	460±6	368±10*	390±6	401±20*	412±18*	<0.0001	<0.0001	0.0020	

**SBP**, Systolic Blood Pressure; **HR**, Heart Rate; **bpm**, beats per minute. Values are mean ± sem. Non-parametric ANOVAs analysis with two factors allowed the evaluation of interaction between aging and genotype. **N** stands for the number of rats; **ns** stands for not significant. Student's T test * p<0.05 to compare SHHF^cp/cp^ *vs.* SHHF^+/?^ at same age.

### Age related arterial stiffening in SHHF rats

No differences in the arterial diameters at systole, diastole and mean BP were detected between the two rat groups either in younger or in older animals (Table 4). The distensibility-pressure curve at 14 months of age for SHHF^+/?^ rats was shifted down words as compared to that of the SHHF^+/?^ animals at 1.5 months of age reflecting stiffening of the carotid during aging (Figure 4B). Similarly, the distensibility-BP curve of the 14-month-old SHHF^cp/cp^ rats was shifted down words but as well to the right in the prolongation of the curve observed in the aged-matched SHHF^+/?^ attesting of higher systolic blood pressure in SHHF^cp/cp^ rats (Figure 4A). Interestingly, at both studied time-points, the values of distensibility at the MBP for the SHHF^cp/cp^ group were significantly decreased as compared to SHHF^+/?^ rats (Table 4, p<0.05) suggesting an altered functionality of the carotid occurring as early as 1.5 months of age. The Einc values were slightly higher in 1.5 month-old SHHF^cp/cp^ animals and significantly increased at 14 months of age (Table 4). Furthermore, the intrinsic mechanical behavior of the wall material evaluated by the wall stress/Einc curve (Figure 4C) and of the wall stress at fixed 600 kPa Einc were decreased with age in all animals but were not significantly different between the two rat groups at any time (Figure 4D). Similarly, the influence of age was detected in the MCSA values (increasing from 1.5 to 14 months of age, p<0.00001), but no association with the genotype could be revealed at any of the two studied time points (Table 4). Overall, the decreased distensibility observed systematically in SHHF^cp/cp^ rats was not associated with dramatic remodeling of their carotid wall over time that could distinguish them from the SHHF^+/?^ rats.

## Discussion

It is now well established that metabolic disorders may dramatically affect heart disease manifestation, especially in the context of a metabolic syndrome when multiple disorders such as obesity, diabetes and dyslipidemia occur simultaneously [2], [3], [16]. There is growing evidence that alterations associated with obesity are not restricted to adipose tissue, but also affect other organs such as brain, liver, and skeletal muscle, resulting in systemic insulin resistance, inflammation, and oxidative stress [9] eventually leading to endothelial and cardiac dysfunction.

Interestingly, different strains of rat develop abnormalities quite similar to those present in patients with MetS and/or obesity [24]. Among them, the SHHF rat is a particularly interesting study model since it spontaneously develops HF either in the presence or absence of MetS [12], [21] unlike other similar animal models described so far [13]. With the aim to investigate in-depth the impact of MetS on the progression towards cardiac remodeling and subsequent failure, we performed a comprehensive analysis of the SHHF^+/?^ and SHHF^cp/cp^ rats phenotypes at cardiac, renal and vascular levels. The concomitant phenotyping of both lean and obese rats helped us refine the physiopathological status of the model during development of HF and clarify discrepancies reported previously for the SHHF model. The side-by-side comparison for a period of 12.5 months (1.5 to 14 months of age) of the SHHF^cp/cp^ and SHHF^+/?^ rats allowed us to characterize the sequence of events leading towards the faster development of heart failure in the obese rats.

If phenotypically barely distinguishable at 1.5 month-old, the SHHF^+/?^ and SHHF^cp/cp^ rats develop very distinctive phenotypes with age. As reported previously SHHF^cp/cp^ rats have a shorter life expectancy than their SHHF^+/?^ littermates (data not shown). This might be explained by the development of severe metabolic disorders that is exclusively present in the obese rats and consequently affected pejoratively their cardiac and renal functions.

Interestingly, altered serum lipidic profiles, presence of insulin resistance and higher adiponectin levels accompanied with hyperaldosteronism were found in young SHHF^cp/cp^ animals (1.5 month-old). The contribution of each of these metabolic factors in obesity and/or MetS development is well known [25], [26], and it is conceivable that their alteration with ageing together with the hyperphagia resulting from the leptin receptor inactivation, participates in the development of the massive obesity and non-alcoholic hepatic steatosis found in SHHF^cp/cp^ rats. Since the metabolic disorders arise at 1.5 months of age when cardiac function and blood pressure were not different between the genotypes, it is likely that these deregulations may have participated in the faster cardiac function decline observed in the SHHF^cp/cp^ rats.

In discordance with reports indicating that the obese SHHF rats are affected by diabetes [13], [27] we monitored glucose concentrations in blood and urine during aging in both groups of rats and never observed fasting hyperglycemia or glycosuria. However, high levels of fasting serum insulin in the SHHF^cp/cp^ rats reflecting the development of an insulin resistance, rather than type 2 diabetes were detected as early as 1.5 months of age. Although SHHF^cp/cp^ rats did not develop diabetes, they presented polydipsia and polyuria that were not associated with dramatic histological alteration of the kidney at the earliest studied age. Despite the absence of glycosuria, interestingly renal histological analysis of 14 month-old SHHF^cp/cp^ rats showed renal lesions similar to those described for diabetes, i.e. hypercellularity, glomerular sclerosis, and increased glomerular surface. The massive proteinuria observed at 5 months of age in SHHF^cp/cp^ rats was consistent with previous reports [17]. Thus, our data suggest that the SHHF^cp/cp^ rats exhibit pre-diabetic features rather than diabetic type 2 trademarks. Since the SHHF strain originates from the breeding of SHR/N-cp rats themselves derived from the original Koletsky rat colony, with SHR-N rats [14] they are likely to share unidentified protective genes that may protect against the onset of Type 2 diabetes in the face of extreme obesity and insulin resistance proposed to be also present in the SHROB genetic background. Indeed, diabetes is not an intrinsic function of the cp mutation itself but likely requires polygenic interaction with other diabetogenic modifier genes present in the background of other strains [28]. Of note, SHHF^cp/cp^ rats did not reach end-stage renal disease causing the reduction of urine volume by the time our protocol ended i.e. at 14 months of age. It is noteworthy that, like dyslipidemia, alterations in the kidney function have been described as risk factors favoring the development of HF, rendering the SHHF strain an adequate model to study the implication of MetS in the decline of the cardiac function. While the discrepancies regarding the diabetic status of SHHF rats requires further analysis, the combination of hyperlipidemia and a pre-hypertensive state as early as 1.5-months of age in the SHHF^cp/cp^ rats nevertheless demonstrates two critical hallmarks of MetS. By 5 months of age the obesity was established in SHHF^cp/cp^ determining the onset of the MetS only in this genotype.

The concurrent comparison of cardiac remodeling between the SHHF^+/?^ and SHHF^cp/cp^ groups of rats allowed us to confirm data from previous reports [15], [20], [21] and extend further the knowledge about the consequences of metabolic disorders on the heart.

At 1.5 months of age, the echocardiographic phenotyping could not distinguish the two rat strains but the simultaneous evaluation of cardiac function in both SHHF^+/?^ and SHHF^cp/cp^ rats during aging indicated that animals differ by the type of cardiac remodeling they develop. The left ventricular wall remodeling is hypertrophic in both groups but is eccentric in SHHF^cp/cp^ while it is concentric in SHHF^+/?^ rats at 14 months of age. Indeed the LV diastolic diameter is greater in SHHF^cp/cp^ rats from 6 months to 14 months of age when the LV internal cavity expands dramatically. Together with the differential modulation of E and A velocity waves between the genotypes over time, cardiac remodeling observed in the SHHF^cp/cp^ group is characteristic of cardiac diastolic dysfunction.

The premature sudden deaths observed during the phenotyping of the SHHF^cp/cp^ group (4 deaths out of 9 SHHF^cp/cp^ rats involved in the follow-up protocol) precluded the observation of a fully declined systolic function at 14 months of age. However, the systolic function evaluation throughout the follow-up of ejection and shortening fractions indicated that those parameters were significantly lower in SHHF^cp/cp^ than in SHHF^+/?^ rats. Moreover, this difference increased significantly after 6 months of SHHF^cp/cp^ rat survival, reflecting the onset of a cardiac decompensation state. By 14 months, SHHF^cp/cp^ animals exhibited dilation of their LV, concomitantly with depletion of the walls and a drop in EF and FS values. Unlike previous reports [13], [18], we did not observe any congestion in the heart of SHHF^cp/cp^ animals, neither during the compensatory cardiac remodeling phase (before 6 months) nor during the decompensate phase (after 6 months of age). However, a massive congestion was observed in 22 month-old lean SHHF rats from an unrelated series of animals (not shown). Altogether our results show that SHHF rat strains exhibit rather specific features stressing the importance and relevance of studying the signaling pathways specifically stimulated or muted during the development of heart failure phenotype associated with each etiology.

Arterial stiffening resulting from metabolic injury or natural aging is a mechanism that might accelerate cardiac remodeling [29], [30]. The deleterious implication of the metabolic disorders in altering hemodynamic parameters was also suggested in other experimental models. Among others, Sloboda et al [31] demonstrated that in old obese Zucker rats had elevated plasma free fatty acid levels alter arterial stiffness was a result of endothelial dysfunction and higher systolic arterial pressure. Based on those data, it is conceivable that the early increase in FFA observed in SHHF^cp/cp^ rats might participate in the impairment of carotid distensibility and compliance in these animals while no difference in the echocardiographic parameters could yet be detected. SHHF^cp/cp^ rats exhibited higher FFA than that of SHHF^+/?^ counterparts but also developed higher blood pressure overtime. For the first time, we evaluated the mechanical properties of the carotid artery in SHHF^cp/cp^ as compared with SHHF^+/?^ rats when only few metabolic disorders were established (1.5 month), and during the decline of cardiac function (14 months of age). Altogether, the significant alteration of carotid distensibility observed in SHHF^cp/cp^ rats suggested that dyslipidemia together with hypertension conjointly affected the mechanical properties of the arteries as early as 1.5 months of age.

While our findings were obtained from a longitudinal study design, they are based on a relatively small sample size that did not allow the sacrifice of animals at intermediary time points. However, the SHHF^cp/cp^ rats still alive at 14 months of age certainly showed the less severe symptomatology as compared to the rats which died prematurely. This probably introduced a bias in our data analysis by minimizing the significance of the differences observed between the SHHF^+/?^ and SHHF^cp/cp^ groups.

As it is not yet clear whether diastolic heart failure progresses towards systolic heart failure or if both, diastolic and systolic dysfunctions are two distinct manifestations of the large clinical spectrum of this disease, there is a clear interest for experimental models such as the SHHF rat. Because alterations of the filling and of the contraction of the myocardium were observed in the SHHF rats, a further refined comparison of the myocardial signal pathways between obese and lean could help discriminating the common physiopathological mechanisms from the specific ones. The echographic manifestation of telediastolic elevation of left ventricular pressure (lower IVRT and increase of E/e' ratio) reflects the altered balance between the preload and afterload of the heart, which are a paraclinical early signs of congestion. These measurements and evaluation are routinely performed during the follow-up of HF human patients.

Several clinical manifestations described in congestive heart failure patients were not observed in the SHHF^cp/cp^ rats but it is likely that the massive obesity in these animals modified profoundly their appearance that might have hidden the manifestation of oedema. Nevertheless, the hyperaldosteronism is in favour of the development of hydrosodic retention in this experimental model. A phenotypic evaluation of older rats might have allowed the observations of fully developed congestive heart failure as it has been reported by others, knowing that congestion is one of the latest clinical phenotypes appearing in humans. The high levels of hormone secretions such as aldosterone are known also in humans to affect the myocardium by causing at least fibrotic remodelling over the long term. The hyperaldosteronism developed by the SHHF rats makes this model appropriate to study the influence of the renin angiotensin aldosterone system on heart failure progression.

Furthermore, the SHHF^cp/cp^ rat allows the study of comorbid conditions like renal dysfunction, insulin resistance, obesity, dyslipidaemia, hypertension that have been pinpointed as major determinants of outcomes in patients with HF. The apparent conflicting results demonstrating that unlike Zucker and Koletsky rats, obese SHHF^cp/cp^ rats develop elevated serum adiponectin levels, which might in fact reinforce the pathophysiological pertinence of this latter strain from a cardiovascular point of view. Recent studies in human have described that in contrast with patients « solely » at risk of cardiovascular disease, circulating adiponectin levels are increased in patients with chronic heart failure, and this finding is associated with adverse outcomes [32]. Furthermore a concept has emerged of functional skeletal muscle adiponectin resistance that has been suggested to explain the compensatory elevated adiponectin levels observed in chronic heart failure [33]. Contrary to Zucker and Kolestky rats which develop mainly hypertension-induced heart dysfunction rather than heart failure, SHHF rats have the advantage to develop spontaneously HF with elevated serum adiponectin levels. Although a more detailed comparison of all these animal models is required, SHHF strains seem more appropriate to study HF than the other strains as they better mimic the human heart failure condition.

Altogether, our results reinforce and complete those obtained by others that conclude on the pertinence of the SHHF rat model in studying the impact of MetS on HF [13], [15], [24], [34], [35], [36]. The SHHF model is an instrumental model to address those issues first, because the metabolic alterations appear in the animals at a very early stage (1.5 months of age); second, because all the rats develop cardiac remodeling, and third, because the severity of the cardiac phenotypes mirror the worsening of metabolic parameters. Importantly, and unlike other animal disease models developing a MetS *per se* (Obese prone rat, SHROB for example) the SHHF strain does develop HF spontaneously [24]. Even though some other strains like the ZSF1 strain [37] could be interesting to study cardiovascular complications of MetS, they develop such dramatic renal injury that the animals die from kidney failure or urinary track pathologies before the establishment of HF features. This is a clear advantage of the SHHF strain over others for studying the biological mechanisms underlying the progression from MetS-induced metabolic disorders to cardiac remodeling.

Here we describe several non-mutually exclusive physiological alterations (dyslipidemia, obesity, renal dysfunction, hypertension, arterial stiffness) that individually, but concomitantly, participate to the adverse cardiac effects of MetS. Since these alterations are established long before their cardiac consequences are detectable in SHHF^cp/cp^ rats, the first trimester of the rat's life appears as an optimal time window for evaluating preventive treatment strategies in these animals.

As suggested in the present study, the pathological molecular programming of cardiac and vascular remodeling had to occur before the detection of their consequences on the phenotypes. An early intervention on MetS-associated disorders may have the potential to prevent, delay or mitigate the renal and vascular alterations as well as cardiac remodeling that appear later.
